# Hexavalent Chromium Removal by a *Paecilomyces* sp. Fungal Strain Isolated from Environment

**DOI:** 10.1155/2010/676243

**Published:** 2010-06-07

**Authors:** Juan F. Cárdenas-González, Ismael Acosta-Rodríguez

**Affiliations:** Laboratorio de Micología Experimental, Centro de Investigación y de Estudios de Posgrado, Facultad de Ciencias Químicas, Universidad Autónoma de San Luis Potosí, Avenue Dr. Manuel Nava No. 6, Zona Universitaria, 78320 San Luis Potosí, SLP, Mexico

## Abstract

A resistant and capable fungal strain in removing hexavalent chromium was isolated from an environment near of Chemical Science Faculty, located in the city of San Luis Potosí, Mexico. The strain was identified as *Paecilomyces* sp., by macro- and microscopic characteristics. Strain resistance of the strain to high Cr (VI) concentrations and its ability to reduce chromium were studied. When it was incubated in minimal medium with glucose, another inexpensive commercial carbon source like unrefined and brown sugar or glycerol, in the presence of 50 mg/L of Cr (VI), the strain caused complete disappearance of Cr (VI), with the concomitant production of Cr (III) in the growth medium after 7 days of incubation, at 28°C, pH 4.0, 100 rpm, and an inoculum of 38 mg of dry weight. Decrease of Cr (VI) levels from industrial wastes was also induced by *Paecilomyces* biomass. These results indicate that reducing capacity of chromate resistant filamentous fungus Cr (VI) could be useful for the removal of Cr (VI) pollution.

## 1. Introduction

Chromium (Cr) toxicity is one of the major causes of environmental pollution emanating from tannery effluents. This metal is used in the tanning of hides and leather, the manufacture of stainless steel, electroplating, textile dyeing, and as a biocide in the cooling waters of nuclear power plants, resulting chromium discharges causing environmental concerns [1]. Cr exists in nine valence states ranging from −2 to +6. Of these states, only hexavalent [Cr (VI)] and trivalent chromium [Cr (III)] have primary environmental significance because they are the most stable oxidation forms in the environment [2]. Both are found in various bodies of water and wastewaters [3]. Cr (VI) typically exists in one of these two forms: chromate (CrO_4_^−2^) or dichromate (Cr_2_O_7_^−2^), depending on the pH of the solution [3]. These two divalent oxyanions are very water soluble and poorly adsorbed by soil and organic matter, making them mobile in soil and groundwater [2]. Both chromate anions represent acute and chronic risks to animals and human health, since they are extremely toxic, mutagenic, carcinogenic, and teratogenic [4]. In contrast to Cr (VI) forms, the Cr (III) species: predominantly hydroxides, oxides, or sulphates, are less water soluble, mobile (100 times less toxic) [5], and (1000 times less) mutagenic [6]. The principal techniques for recovering or removing Cr (VI), from wastewater are chemical reduction and precipitation, adsorption on activated carbon, ion Exchange, and reverse osmosis, in a basic medium [7]. These methods present high cost, low efficieny and the toxic sludge generation, which could require to imply disposal complex operations [8].

The ability of some microorganisms to interact with different Cr forms makes them attractive in the context of environmental biotechnology. In this sense, the use of microbial biomass for the removal of Cr from industrial wastewater and polluted water has already been recognized [9]. The properties of some microorganisms to both tolerate and reduce Cr (VI) enable their application in biotechnological process focusing on detoxification of Cr (VI). Cr resistance has been described in bacteria and fungi isolated from Cr-polluted environments. Yeast strains isolated include *Candida* and *Rhodosporidium* genera, but in these, the general mechanism of chromate resistance is related to limited ion uptake, rather than to chemical reduction of the toxic species [10]. However, other yeasts such as *Candida utilis* [11] and *Candida maltosa* [12] showed partial ability to reduce Cr (VI) and also the capability to accumulate Cr in the biomass. Recent reports have also examined Cr (III) and Cr (VI) uptake and accumulation by different filamentous fungi [13, 14]. The present study reports the isolation and identification of a *Paecilomyces *sp. fungal strain that exhibits high level Cr (VI) resistance and reduction potential.

## 2. Experimental

### 2.1. Microorganism and Chromate Resistance Test

A chromate-resistant filamentous fungus was isolated from polluted air with industrial vapors, near of Chemical Science Faculty, located in the city of San Luis Potosí, Mexico, in Petri dishes containing modified Lee's minimal medium [LMM, 15] [with 0.25% KH_2_PO_4_, 0.20% MgSO_4_, 0.50% (NH_4_)_2_SO_4_, 0.50% NaCl, 0.25% glucose] supplemented with 500 mg/L K_2_CrO_4_; the pH of the medium was adjusted and maintained at 5.3 with 100 mMol/L citrate-phosphate buffer. The cultures were incubated at 28°C for 7 days. The strain was identified based on their morphological structures such as color, diameter of the mycelia, and microscopic observation of formation of spores [15]. Chromate-resistant tests of the isolated strain, filamentous fungus *Paecilomyces *sp., were performed on liquid LMM containing the appropriate nutritional requirements and different concentrations of Cr (VI) (as potassium chromate), and determining the dry weight.

### 2.2. Culture Conditions in Liquid Media

Cultures in 100 mL of sterile LMM [amended with 50 mg/L Cr (VI)] inoculated with 5 × 10^5^ spores/mL were incubated at 28°C for 48 hours. Then, cells were aseptically separated by centrifugation at 2000 rpm (4°C) for 10 minutes, and washed twice with sterile trideionized water to eliminate culture medium components and cell debris. The cell pellet was resuspended in 3 mL of sterile trideionized water by shaking in a vortex mixer for 30 seconds, and was then transferred to 100 mL of fresh LMM amended with 50 mg/L Cr (VI). At various times during the course of incubation, 1 mL aliquots were removed and centrifuged at 5000 rpm for 10 minutes to sediment the cells; the supernatant fluid was used to determine the concentration of Cr (VI) or total Cr.

### 2.3. Determination of Hexavalent, Trivalent, and Total Cr

Hexavalent Cr and trivalent chromium were quantified by a spectrophotometric method employing diphenylcarbazide and chromazurol S, respectively [16, 17], total Cr was determined by electrothermal atomic absorption spectroscopy [16]. 

The values shown in Section 3 are the mean from three experiments carried out by triplicate.

## 3. Results and Discussion

### 3.1. Isolation and Identification of a Fungal Strain Capable of Removing Cr (VI)

**Figure 1:**
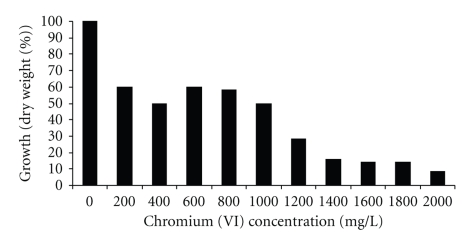
Growth in dry weight of *Paecilomyces *sp. with different concentrations of Cr (VI). 1 × 10^5^ spores/mL, 28°C, 7 days of incubation, 100 rpm.

The fungal strain isolated was able to grow on LMM supplemented with 2000 mg/L of Cr (VI) (Figure 1). This indicates that this fungus developed the Cr (VI) resistance and probably the Cr (VI) is being reduced in the polluted air. A variety of microorganisms with the Cr (VI) resistance and Cr (VI) reducing ability have been isolated from effluents of tanneries [3, 7, 14]. Colonies of the isolated fungal strain grew rapidly and mature within 3 days. *Paecilomyces *sp. are thermopile and can grow well at temperatures as high as 50° and 60°C. The colonies are flat, powdery, or velvety in texture. The initial color is white, and becomes yellow, yellow-green, pink, or violet. The reverse is dirty white or buff. A sweet aromatic color may be associated with older cultures. Septate hyaline hyphae, conidiophores, phialides, conidia, and chlamidospores are observed. Conidiophores (3-4 *μ*m wide and 400–600 *μ*m long) are often branched and carry the phialides at their tips. The phialides are swollen at their bases and taper towards their apices. They are usually grouped in pair or brush-like clusters. Conidia are unicellular, hyaline to darkly colored, smooth or rough, oval to fusoid, and form long chains. Chlamidospores are occasionally present [15].

### 3.2. Effect of pH

**Figure 2:**
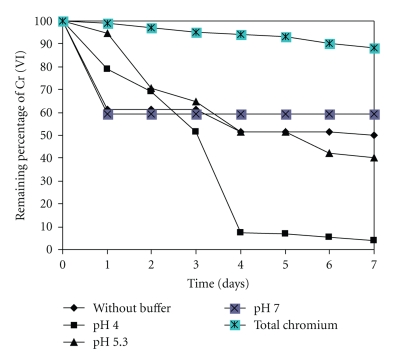
The effect of pH on Chromium remotion by *Paecilomyces *sp. 50 mg/L Cr (VI), 100 rpm, 28°C.

Figure 2 shows the effect of varying pH (4.0, 5.3, and 7.0, maintained with 100 mmol/L citrate-phosphate buffer.) on the rate of Cr (VI) removal. The rate of chromium uptake and the extent of that capture were enhanced as the pH falls from 7.0 to 4.0. The maximum uptake was observed at pH 4.0 (96% at 7 days), 96%, Liu et al. and Bai and Abraham [18, 19] reported maximum removal at 100 mg/L Cr (VI) solution using *Mucor racemosus *and *Rhizopus nigricans* with pH optimum of 0.5–1.0 and 2.0, respectively, Sandana Mala et al. [20], at pH 5.0 for Cr (VI) with *Aspergillus niger* MTCC 2594, Rodríguez et al. [21], at pH 3.0–5.0 for Pb^+2^, Cd^+2 ^and Cr^+3  ^with the yeast *Saccharomyces cerevisiae*, Park et al. [22], at pH 1–5 for Cr (VI) with brown seaweed *Ecklonia*, Higuera cobos et al. [23], at pH 5.0 for Cr (VI) with the brown algae *Sargassum *sp, and Fukuda et al. [14], at pH 3.0 for Cr (VI) with *Penicillium* sp. In contrast to our observations, Prasenjit and Sumathi [24] reported maximum uptake of Cr (VI) at pH 7.0 with *Aspergillus foetidus*, Puranik and Paknikar [25] reported an enhanced uptake of lead, cadmium, and zinc, with a shift in pH from 2.0 to 7.0 using a *Citrobacter *strain, and a decrease at higher pH values. Al-Asheh and Duvnjak [26] also demonstrated a positive effect of increasing pH in the range 4.0–7.0 on Cr (III) uptake using *Aspergillus carbonarius*. At low pH, the negligible removal of chromium may be due to the competition between hydrogen (H+) and metal ions [27]. At higher pH (7.0), the increased metal removal may be due to the ionization of functional groups and the increase in the negative charge density on the cell surface. At alkaline pH values (8.0 or higher), a reduction in the solubility of metals may contribute to lower uptake rates.

### 3.3. Effect of Cell Concentration

**Figure 3:**
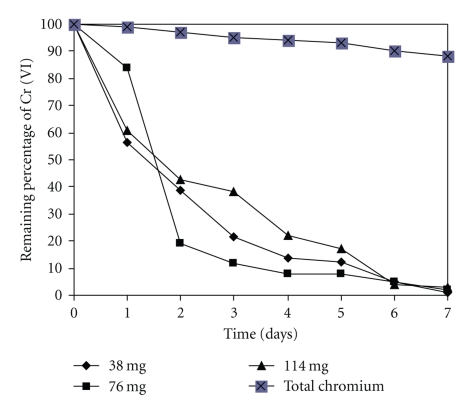
The effect of cell concentration on the removal of Cr (VI). 50 mg/L Cr (VI). 100 rpm, 28°C, pH 1.0.

The influence biomass in the removal capacity of Cr (VI) was depicted in Figure 3. From the analyzed 38, 76, and 114 mg of dry weight the removal capacity was in the order of 99.17%, 97.95%, and 97.25%, respectively. In contrast to our observations, most of the reports in the literature observe at higher biomass dose resulting in an increase in the percentage removal [1, 3, 7, 8, 18, 21]. The higher the biomass dose, the more binding sites for complex of Cr (VI) (e.g., HCrO_4_^−^ and Cr_2_O_7_^−2^ ions) [3, 9]. However, it did not show in our observations.

### 3.4. Effect of Initial Cr (VI) Concentration

**Figure 4:**
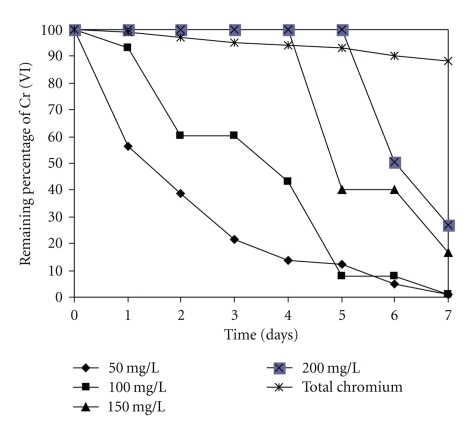
The effect of the concentration of Cr (VI) in solution on the removal. 100 rpm, 28°C, pH 4.0.

As seen in Figure 4, when the initial Cr (VI) ions concentration increased from 50 mg/L to 200 mg/L, the percentage removal of metal ions decreased. This was due to the increase in the number of ions competing for the available functions groups on the surface of biomass. Our observations are like most of the reports in the literature [1, 3, 5, 7, 8, 18, 21–24].

### 3.5. Effect of Carbon Source

**Figure 5:**
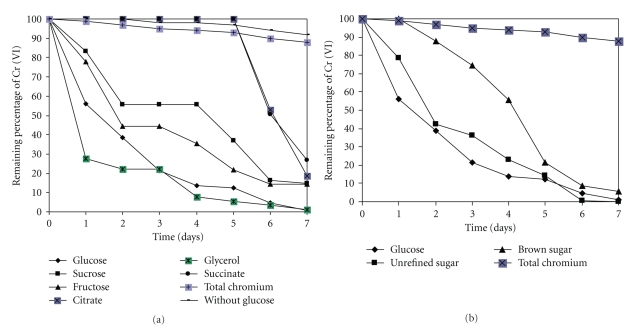
Influence of carbon source on the capability of *Paecilomyces *sp. to decrease Cr (VI) levels in the growth medium. 100 rpm, 28°C, pH 4.0. Influence of commercial carbon sources and salt on the capability of *Paecilomyces *sp. to decrease Cr (VI) levels in the growth medium. 100 rpm, 28°C, pH 4.0.

Figures 5(a) and 5(b) show that the decrease of Cr (VI) level in culture medium of *Paecilomyces *sp. occurred exclusively in the presence of a carbon source, either fermentable (glucose sucrose, fructose, citrate) or oxidable (glycerol). In the presence of glucose, another inexpensive commercial carbon sources like unrefined sugar and brown sugar or glycerol, the decrease in Cr (VI) levels occurred at a similar rate, at 7 days of incubation, are of 99.17%, 100%, 94.28%, 81.5, and 99%, respectively, and the other carbon sugar were less effectives. On the other hand, incubation of the biomass in the absence of a carbon source did not produce any noticeable change in the initial Cr (VI) concentration in the growth medium. These observations indicated that in culture of the fungus a carbon source is required to provide the reducing power needed to decrease Cr (VI) in the growth medium. Our observations are like to the report of Acevedo Aguilar et al., Prasenjit and Sumathi [13, 24] with glucose like carbon source, and are different from the observations of Srivasta and Thakur [28] with *Aspergillus* sp, and *Acinetobater* sp, who observed how the main carbon source is the sodium acetate.

### 3.6. Time Course of Cr (VI) Decrease and Cr (III) Production

**Figure 6:**
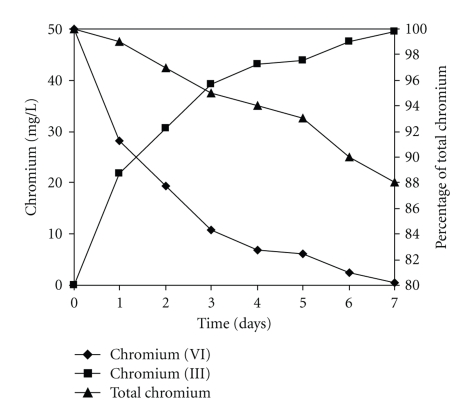
Time-course of Cr (VI) decrease and Cr (III) production in the spent medium of culture initiated in Lee's minimal medium, amended with 50 mg/L Cr (VI). 100 rpm, 28°C, pH 4.0.

The ability of the isolated strain to lower the initial Cr (VI) of 50 mg/L and Cr (III) production in culture medium was analyzed. Figure 6 shows that *Paecilomyces *sp. exhibited a remarkable efficiency to diminish Cr (VI) level with the concomitant production of Cr (III) in the growth medium. Thus, after 7 days of incubation, the fungus strain caused a drop in Cr (VI) from its initial concentration of 50 mg/L to almost undetectable levels, and the decreased level occurred without significant change in total Cr content. As expected, total Cr concentration remained constant over time, in medium without inoculum. These observations indicate that *Paecilomyces *sp. strain is able to reduce Cr (VI) to Cr (III) in growth medium amended with chromate. There are two mechanisms by which chromate could be reduced to a lower toxic oxidation state by an enzymatic reaction. Currently, we do not know whether the fungal strain used in this study expresses Cr (VI) reducing enzyme(s). Further studies are necessary to extend our understanding of the effects of coexisting ions on the Cr (VI) reducing activity of the strain reported in this study. Cr (VI) reducing capability has been described in some reports in the literature [2, 8, 11–14, 19, 20, 22, 24, 27]. Biosorption is the second mechanism by which the chromate concentration could be reduced, and 1 g of fungal biomass of *Paecilomyces *sp. is able to remove 1000 mg/L of Cr (VI) at 60°C, at 3 hours of incubation (date not shown), because the fungal cell wall can be regarded as a mosaic of different groups that could form coordination complexes with metals, and our observations are like most of the reports in the literature [1, 3, 5, 7–9, 18, 21–24].

### 3.7. Removal of Cr (VI) in Industrial Wastes with Fungal Biomass

**Figure 7:**
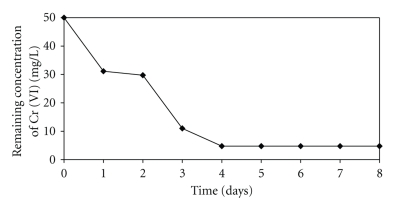
Removal of Chromium (VI) in industrial wastes incubated with the fungal biomass. 100 rpm, 28°C, pH 4.0, 50 g of contaminated soil (50 mg Cr (VI)/g soil).

We adapted a water-phase bioremediation assay to explore possible usefulness of strain of *Paecilomyces *sp., for eliminating Cr (VI) from industrial wastes, the mycelium biomass was incubated with nonsterilized contaminated soil containing 50 mg Cr (VI)/g, suspended in LMM, pH 4.0. It was observed that after eight days of incubation with the *Paecilomyces *sp. biomass, the Cr (VI) concentration of soil sample decreased fully (Figure 7), and the decreased level occurred without significant change in total Cr content, during the experiments. In the experiment carried out in the absence of the fungal strain, the Cr (VI) concentration of the soil samples decreased by about of 18% (date not shown); this might be caused by indigenous microflora and (or) reducing components present in the soil. The chromium removal abilities of *Paecilomyces *sp. are equal or better than those of other reported strains, for example, *Candida maltose* RR1 [12]. In particular, this strain was superior to the other strains because it has the capacity for efficient chromium reduction under acidic conditions. Most other Cr (VI) reduction studies were carried out at neutral pH [14, 15]. *Aspergillus niger* also has the ability to reduce and adsorb Cr (VI) [14]. When the initial concentration of Cr (VI) was 500 ppm, *A. niger* mycelium removed 8.9 mg of chromium/g dry weight of mycelium in 7 days. In the present study, *Paecilomyces *sp., remove 50 mg/g, (pH, 4.0 and 8 days).

## 4. Conclusion

The *Paecilomyces *sp. fungal strain was isolated from polluted air with industrial vapors. This strain showed the capacity at complete concentrations reduction of 50 mg/L Cr (VI) in the growth medium after 7 days of incubation, at 28°C, pH, 4.0, 100 rpm, and an inoculum of 38 mg of dry weight. These results suggest the potential applicability of *Paecilomyces *sp. for the remediation of Cr (VI) from polluted soils in the fields.
